# Trauma and mental health of medics in eastern Myanmar’s conflict zones: a cross-sectional and mixed methods investigation

**DOI:** 10.1186/1752-1505-7-15

**Published:** 2013-07-30

**Authors:** Andrew George Lim, Lawrence Stock, Eh Kalu Shwe Oo, Douglas P Jutte

**Affiliations:** 1 UC Berkeley-UCSF Joint Medical Program at the University of California, Berkeley School of Public Health, 570 University Hall #1190, Berkeley, CA 94720, USA; 2 University of California, San Francisco School of Medicine, 500 Parnassus Ave., San Francisco, CA 94143, USA; 3 Global Health Access Program, 22/13 Trok Naikao, Mae Sot, Tak 63110, Thailand; 4 David Geffen School of Medicine at the University of California, Los Angeles, 924 Westwood Blvd., Suite 300, Los Angeles, CA 90024, USA; 5 Karen Department of Health and Welfare, 9/164 Soi 3 Mae Sot Villa, Intarakiri Rd., Mae Sot, Tak 63110, Thailand; 6 Present address: 2550 Ninth St., Suite III, Berkeley, CA 94110, USA

**Keywords:** Burma, Myanmar, Trauma, Mental health, PTSD, Conflict, Healthworkers, Medics, Anxiety, Depression, Burnout, Vicarious traumatization

## Abstract

**Background:**

In conflict and disaster settings, medical personnel are exposed to psychological stressors that threaten their wellbeing and increase their risk of developing burnout, depression, anxiety, and PTSD. As lay medics frequently function as the primary health providers in these situations, their mental health is crucial to the delivery of services to afflicted populations. This study examines a population of community health workers in Karen State, eastern Myanmar to explore the manifestations of health providers’ psychological distress in a low-resource conflict environment.

**Methods:**

Mental health screening surveys were administered to 74 medics, incorporating the 12-item general health questionnaire (GHQ-12) and the posttraumatic checklist for civilians (PCL-C). Semi-structured qualitative interviews were conducted with 30 medics to investigate local idioms of distress, sources of distress, and the support and management of medics’ stressors.

**Results:**

The GHQ-12 mean was 10.7 (SD 5.0, range 0–23) and PCL-C mean was 36.2 (SD 9.7, range 17–69). There was fair internal consistency for the GHQ-12 and PCL-C (Cronbach’s alpha coeffecients 0.74 and 0.80, respectively) and significant correlation between the two scales (Pearson’s R-correlation 0.47, P<0.001). Qualitative results revealed abundant evidence of stressors, including perceived inadequacy of skills, transportation barriers, lack of medical resources, isolation from family communities, threats of military violence including landmine injury, and early life trauma resulting from conflict and displacement. Medics also discussed mechanisms to manage stressors, including peer support, group-based and individual forms of coping.

**Conclusions:**

The results suggest significant sources and manifestations of mental distress among this under-studied population. The discrepancy between qualitative evidence of abundant stressors and the comparatively low symptom scores may suggest marked mental resilience among subjects. The observed symptom score means in contrast with the qualitative evidence of abundant stressors may suggest the development of marked mental resilience among subjects. Alternatively, the discrepancy may reflect the inadequacy of standard screening tools not validated for this population and potential cultural inappropriateness of established diagnostic frameworks. The importance of peer-group support as a protective factor suggests that interventions might best serve healthworkers in conflict areas by emphasizing community- and team-based strategies.

## Background

### The mental health of medical providers in conflict areas

In conflict settings around the world, medics are dispatched to address the emergent health needs of civilian populations that do not otherwise have access to healthcare. International humanitarian law (IHL) demands protection for patients, medical facilities, and vehicles. It also grants protection for medical personnel so long as they perform their humanitarian duties and do no harm to the enemy [1]. In civil conflicts, rules of medical neutrality are often ignored and medical personnel and their patients become targets [2]. Medical personnel in armed conflicts face chronic stressors and personal risk that can lead to poor mental health outcomes, including post-traumatic stress disorder (PTSD) [3]. In this study, the terms *medic* or *healthworker* refer to non-physician civilian or military personnel responsible for the provision and delivery of health services.

Mental illness is an often-overlooked public health concern in conflict areas. Studies show an increase in the incidence and prevalence of PTSD, anxiety, and depression among civilian populations as a consequence of war [4-7]. Given the high stress environments and low-resource settings in which they operate, medics are at particular risk for developing such mental health disorders [8]. In these demanding environments, health responders unable to cope with significant traumatic stress may develop chronic psychological symptoms and be at increased risk for substance abuse [9]. In the long term, health providers may experience burnout and loss of professional engagement [10]. Ultimately, mental distress can adversely affect health providers’ performance, leading to compromised efficacy in treating target populations [11-13].

Much of the discussion around conflict and mental health has revolved around clinical diagnoses of individuals as outlined in the Diagnostic and Statistical Manual of Mental Health Disorders (DSM) [14-17]. However, psychiatrists, psychologists, anthropologists, and other health professionals working in war-afflicted communities have attempted to broaden the focus of mental health studies beyond the individual’s experience to include socio-cultural determinants of collective suffering [18-23]. Furthermore, it is critical to look beyond DSM causal definitions and explore the mechanisms of human growth, self-transformation, and resilience affecting individual mental health outcomes [24-26].

For these reasons, studies utilizing quantitative mental health assessments that are accompanied by qualitative approaches can explore idioms and manifestations of distress unique to a particular population [27]. Though mental health has been extensively explored among civilian and soldier populations in conflict settings, there is a relative dearth of literature regarding the specific mental distress among health professionals and first responders involved in conflicts [8]. As such, qualitative research can offer insight into the most salient problems for this under-studied population.

### Civil conflict in Burma/Myanmar and community-based health responders

Since 1962, civil war between the government of Myanmar (also known as Burma) and independent ethnic factions including the Karen National Union (KNU) has caused egregious human rights abuses and unmet public health needs for ethnic minority population [28-30]. Even with the cease-fire agreement between the KNU and Burmese government as of January 2012, the legacy of displacement, violence, and forgotten landmines will likely persist for subsequent generations.

Medics trained by community-based health organizations are largely responsible for basic healthcare, disease prevention, and public health education in IDP areas. They work despite profound barriers to service delivery, including under-resourced clinics and limited training capacity for complex medical procedures.

The KNU-affiliated Karen Department of Health and Welfare (KDHW) provides care to 100,000 forcibly-displaced and war-affected residents of Karen State [31,32]. The Back Pack Health Worker Team (BPHWT) serves a target population of 187,000 in the conflict zones of Karen, Karenni, Mon and Shan states [33]. Their teams serve the villages that are most inaccessible, including IDP areas without access to even KDHW mobile health clinics [32].

A medic’s education begins after completion of secondary education (at approximately 17–19 years old). The medics-in-training undergo two years of basic community healthworker training provided by BPHWT or KDHW, and senior medics learn advanced care topics (management of traumatic injuries, basic obstetric care, and infectious disease control, as examples).

These healthworkers persevere despite perilous security threats. Human rights organizations have reported accounts of Burmese soldiers targeting civilian clinics and medic teams [33-35]. Medics can carry small side arms for self-defense purposes only, but otherwise are required to remain non-combatants.

Mental health evaluation studies have been carried out among refugee and immigrant populations living in Thailand near the Burmese border [36-40] and qualitative research has illuminated the challenges to health service delivery in eastern Myanmar [41]. However, no studies have explored the psychological effects of conflict on the health providers working among internally displaced populations (IDP) in this region.

### Study aims

Using a mixed-methods approach, this study examined the local idioms of distress and mental health experiences of mobile medics providing services to war-afflicted communities in eastern Myanmar. This paper aims to elucidate these unique mental health concerns in order to inform the development of provider-focused interventions more broadly.

## Methods

### Study design – a mixed methods approach

This study combined quantitative screening instruments with qualitative open-ended interviews in an effort to triangulate manifestations of psychological distress and trauma among the medics. Two screening tools were used: the Posttraumatic Checklist for civilians (PCL-C), and the 12-question General Health Questionnaire (GHQ-12).

The PCL has been well validated as a self-report instrument to assess symptoms of PTSD based on DSM-IV diagnostic criteria [42]. When responses are answered on a five-point ordinal response scale (1–5), the total score ranges from 17 to 85 (high scores signify severe symptoms). It has demonstrated reliable psychometric properties compared to other well-established self-reporting instruments [43] and correlates well with diagnostic clinician-administered surveys [44]. PCL-C threshold values have been shown to have good diagnostic efficiency with a cutoff of 32 (sensitivity = 0.76, specificity = 0.90) for screening soldiers post-combat in primary care setting [45]. It has also been shown to have a diagnostic efficiency of 0.825 (sensitivity = 0.944, specificity = 0.864) with a cutoff of 44 among civilians in a primary care setting [44], and a diagnostic efficiency of 0.80 (sensitivity = 0.91, specificity = 0.40) among veterans in mental health care-seeking population [46,47].

The GHQ-12 is used to detect non-specific symptoms of psychiatric disorders at a community-level [48]. It has been used to screen symptoms for a range of anxiety and depressive disorders as defined by the DSM-IV, including major depressive disorder, agoraphobia, panic disorder, generalized anxiety disorder, chronic fatigue, and mixed anxiety-depression in populations affected by political violence [49,50]. When responses are answered on a four-point ordinal response scale (0–3), the total score ranges from 0 to 36 (high scores signify severe symptoms). Alternatively, the same GHQ-12 ordinal response scale responses can be scored on a two-point scale (four-point scale responses translate to 0-0-1-1).

The screening instruments were translated into Skaw Karen and blindly back-translated by a second translator. The survey was pre-tested in Mae Sot, Thailand with individuals of comparable education to the medics. Surveys omitted personal identifying information, but included basic demographic data (sex, age, years of experience, marital status).

The authors developed a preliminary, semi-structured guide for the interviews based on literature review and key informant recommendations. The key informants held leadership positions within KDHW or BPHWT and were all former or current medics. The final interview guide aimed to 1) characterize mental health terms commonly used in Burmese and Karen (“local idioms of distress”) and to describe the emotional and physiological manifestations associated with these experiences, 2) characterize the challenges faced by the medics and how personal traumatic events (either during or prior to working as a medic) impacted their psychological well-being, 3) give examples of coping mechanisms used to deal with life stressors, and 4) discuss motivations, goals and other factors contributing to resilience against stressors (see Additional file 1).

### Study setting, participants, and implementation

Data was collected over three medic training courses near Mae Sot and Suan Phung, Thailand between July 2010 and July 2011 (see Table 1). Seventy-four mental health assessments (combined GHQ-12 and PCL-C) were completed by all participants from two trainings. Respondents were instructed to take 30–60 minutes to answer the written survey in a private location of their choosing.

**Table 1:** Summary of study participants

**Training date:**	**Jul-Aug 2010**	**Jan 2011**	**Jun-Jul 2011**	**Total subjects:**
***Training site:***	**Mae Sot**	**Suan Phung**	**Mae Sot**	
***Organization:***	**BPHWT**	**KDHW**	**BPHWT**	
*# medics present at training:*	22	45	29	
*# qualitative interviews conducted:*	15	15	0	**30**
*# mental health assessments administered (GHQ-12* &*PCL-C):*	0	45	29	**74**

A total of 30 qualitative interviews were conducted via chain sampling. Twenty-seven interviews required the assistance of an interpreter, with medics’ responses translated from Skaw Karen (and Burmese in one case) to English. Selection criteria prioritized subjects with more than one year of current field experience.

With the informed consent of participants, interviews and surveys were conducted with maximal attention to privacy at training locations or personal quarters. All interviews were audio-recorded and English translations were used for analysis. All identifying information of interviews and surveys were omitted.

The project was discussed with local leaders to gain support for the study at each training location. The University of California, Berkeley Committee for the Protection of Human Subjects approved this study.

### Quantitative analysis

The subjects’ demographic characteristics and itemized GHQ-12 and PCL-C responses were tabulated using Microsoft Excel 2011 and analyzed with Stata SE v10 [51]. Survey scores were aggregated and population means, medians, and standard deviations were determined. The GHQ-12 (four-point and two-point scoring systems) and PCL-C were tested for internal consistency by calculating Cronbach’s alpha coefficients. The relationships between scores on each scale were evaluated using Pearson’s r-correlations. The authors attempted to control for the effect of medics’ experience level on symptom severity by separating the analysis of experienced medics (defined as two or more years of field experience) from the analysis of all surveyed subjects.

### Qualitative analysis

A grounded theory-based approach to qualitative data analysis was utilized in an attempt to construct theories from which to base future hypothesis-driven research [52]. To this end, the primary author generated theoretical statements from the contextual analysis of participants’ interviews. An initial phase of descriptive coding using HyperRESEARCH v2.6 yielded a total of 677 descriptive codes [53].

These descriptive codes were edited for redundancies, indexed to the source data, and grouped under one of 54 categorical codes. Each categorical code was further organized into one of eight categorical code families, the organization of which formed the basis of the analytical memos. Ultimately, these memos informed the arguments presented in the Discussion section.

The primary author assessed inter-rater reliability of qualitative analysis by randomly selecting interview excerpts to be coded by non-author independent researchers, with reasonable validity of descriptive and categorical codes. Results were member-checked with non-participant medics following the study, showing sufficient transferability. Regarding reflexivity, there may have been investigator biases intrinsic to the collection and interpretation of data, namely the authors’ prior knowledge that significant stressors existed in the population.

## Results

### Demographics

The demographic characteristics of the 74 surveyed respondents and 30 interviewed respondents are shown in Table 2. Participants were nearly equally divided in gender. The surveyed medics were mostly young (45% < 23 years) and under-experienced (35% with less than one year of experience or currently in training). The interviewed medics were slightly older and mostly unmarried, and all had at least two years of field experience except for two new medics in training.

**Table 2:** Demographics

**Variable**	**Mental health surveys (n = 74)**	**%**	**Interview respondents (n=30)**	**%**
*Sex:*				
Male	36	49%	16	53%
Female	38	51%	14	47%
*Age:*				
18-22	33	45%	7	23%
23-27	17	23%	10	33%
28-32	9	12%	7	23%
33-37	5	7%	3	10%
38-42	7	9%	3	10%
43-47	3	4%	0	0%
*Years of experience:*				
1 or less (currently in training)	29	39%	2	7%
2-5	16	22%	10	33%
6-10	18	24%	14	47%
11-15	5	7%	2	7%
16-20	3	4%	2	7%
No response	3	4%	0	0%
*Marital Status:*				
Single	27	36%	22	73%
Married, has children	15	20%	4	13%
No response	32	43%	4	13%

### Quantitative survey results

The 74 medics’ GHQ-12 scores averaged 10.7 (SD 5.0) and 2.5 (SD 2.1) when using the four-point and two-point scales, respectively; the PCL-C scores averaged 36.2 (SD 9.7) (see Table 3). Results showed moderately strong and significant Pearson’s r-correlations between PCL scores and both scoring methods of GHQ-12 (Table 4).

**Table 3:** Mental health survey score distributions

	**All (n =74)**			**Experienced ≥ 2yrs (n = 42)**		
**Survey scale**	**Mean/Median**	**SD**	**Cronbach’s**	**Mean /Median**	**SD**	**Cronbach’s alpha**
	**(Range)**		**alpha**	**(Range)**		
*GHQ-12, four-point scoring (possible score range 0-36)*	10.7 / 11	5.0	0.74	11.2 / 11	4.3	0.70
	(0-23)			(1-20)		
*GHQ-12, two-point scoring (possible score range 0-12)*	2.5 / 2	2.1	0.63	2.2 / 2	2.2	0.65
	(0-8)			(0-8)		
*PCL-C score (possible score range 17-85)*	36.2 / 35	9.7	0.80	36.5 / 36	10.1	0.84
	(17-69)			(17-59)		

**Table 4:** Pearson’s R-correlation coefficients between PCL-C and GHQ-12 (n=74)

**Correlation of PCL-C with:**	**Correlation**	**P-value**
GHQ-12 (0–36 range)	0.4722	<0.00005
GHQ-12 (0–12 range)	0.4109	0.0003

To address the discrepancy in years of experience between the surveyed and interviewed samples, an analysis of the 42 surveyed medics with at least two full years of field experience was done, yielding similar results to the full sample with a GHQ-12 mean of 2.2 (SD: 2.2) and PCL-C mean of 36.5 (SD: 10.1).

Cronbach’s alpha coefficient calculations for all respondents (n=74) yielded values of 0.80, 0.74, and 0.63 for PCL-C, GHQ-12 (four-point scoring), and GHQ-12 (two-point scoring) respectively. This corresponds to good internal consistency for the PCL-C scale, but fair-moderate internal consistency for the GHQ-12 scales.

### Local idioms of distress

Although the majority of interviews were conducted in Skaw Karen, both Burmese and Karen terms were used to describe mental health idioms. Burmese idioms for stress , anxiety , depression , and mental trauma  were more commonly used than their Karen counterparts. Although they are transliterations of Western psychological concepts, the Burmese or Karen terms will be substituted with the English proxies for ease of comprehension throughout this paper. The following describes each local idiom and its relationship to similar terms in English (see Table 5).

**Table 5:** Local idioms of distress

**English**	**Burmese**	**Burmese transliteration**	**Karen**	**Karen transliteration**
Stress		Mind-spirit suppression	*thar law bwi, thar ba hti tor*	Tired heart
Anxiety		Mind-spirit unsteadiness	*thut kot thar gaw*	Liver is hot, heart is red
Depression		Spirit fall	*thar law bwa*	The heart falls down
Mental trauma		Mind injury	*tha ba doe*	Heart-touch hit

*Seik hpizimu* (stress, literally “mind suppression” or “spirit suppression”) was often characterized in terms of physiologic symptoms. Typical associations were heart palpitations, the heart “feeling tired,” an inability or unwillingness to eat, shaking, losing sleep, being antisocial, feeling afraid, having a “heaviness” on the mind, not understanding oneself, thinking too much, or thinking constantly about too many things.

*Seik lo’shaa* (anxiety or worry, literally “mind unsteadiness” or “spirit unsteadiness”) was characterized by perceptions of “falling”, the inability to stand (faintness), palpitations, and shortness of breath.

*Seik da’kya* (depression, literally “spirit fall”) was commonly thought of as a “long-term” ailment “kept in the mind-spirit” (as opposed to the body) associated with trembling, loss of appetite, excessive sleep, social isolation, and feelings of futility.

*Seik daan yaa* (mental trauma, or literally “mind injury”) could be related to either physical injury or verbal abuse. Some medics attributed *seik daan yaa* to hearing stories about attacks resulting in massive casualties in their villages. Medics who had formerly been soldiers mentioned being easily startled by loud noises. Some medics who identified as affected by *seik daan yaa* reported re-experiencing trauma events and having persistent thoughts of death in nightmares. Some medics felt that *seik daan yaa,* if repeated over time, could result in people “losing control of their mind”, or being unable to distinguish reality from imagination or dreams.

### Sources of distress

The interviewed medics discussed a variety of hardships resulting from their roles. These challenges will be discussed as 1) work-related, 2) personal/family-related, 3) related to security, violence, and threats from the Burmese military, and 4) related to early life trauma (see Table 6).

**Table 6:** Summary of medics’ sources of distress (n = 30)

**Work-related challenges**	**Personal/family-related challenges**	**Security issues, conflict, and human rights violations**	**Early life trauma**
Difficulty retaining personnel (11)	Separation from family and/or home village for extended periods of time (24)	Providing medical services in/near areas of active fighting (30)	Early life trauma related to forced displacement (10)
Inability / perceived lack of skills to treat patients (10)	Inability to support family and/or inadequate compensation (8)	Acts of violence against medics, patients and clinics (10)	Early life trauma related to violence against family members (7)
Lack of medical supplies and other resources (10)	Disruption of family due to resettlement issues (4)	Limited ability to provide services due to security risks (9)	
Transportation and distance-related barriers (3)	Fear for family's security (3)	Threat of landmine injury (8)	

### Work-related challenges

Work-related sources of distress resulted from feelings of incompetence, lack of medical resources, transportation barriers and personnel shortages. Additional stressors emerged from the medics’ social interactions within the village communities, where issues of compliance, trust, reputation, and community expectations could strain the medic-patient relationship.

Medics expressed lack of confidence or inadequacy in their ability to manage complex medical situations, even when the necessary resources were available (n=10). Acute trauma due to a landmine blast was the most cited challenging medical situation, with eight medics having had at least one encounter with a landmine victim. Although many medics were able to provide counseling or make referrals to regional clinics, their inability to fulfill patients’ expectations by treating acute ailments was a source of frustration.

The feeling of inadequacy was brought most sharply to focus in situations of patient death. Some described feeling particular distress when a patient died in their care, regardless of whether the medic felt personally at fault (n=6). Even if a patient’s death was unavoidable, medics expressed feeling obligation and responsibility to the patients and their families. In some cases, the patients’ families blamed medics for patient deaths that the medic believed to be inevitable:

I will give an example about two children that I treated. They had malarial shock, and were hypotensive – So, I know they came to me very late [in their illness]. The first one, I treated for five days, and she became better. But, the other one, I knew before he arrived here, the time was very late for him. He was already in shock. After two days of treatment, he died.

So, the family was not happy with me. I wasn’t happy also. At that time I felt sad, because the family told me many things, like I made a mistake. - 26 year-old, male medic

Medics who spoke of feelings of ineptness in patient care also expressed feelings of shame, helplessness, and embarrassment. They were preoccupied by others’ (medics, patients, or community members) opinions of their legitimacy and effectiveness as health providers. Medics often said they felt burdened by the social responsibility of patient care and the reactions of patients they were unable to help:

For example… when we are in the field, treating the people, if we can cure them, they will say thank you. But if we cannot, they will swear [at] you. So, when I cure 20 people, if 10 people do not [become] well, I feel – why [does] it [happen] like this? I have to think: men die, because of me, or because why? [I think about] this a lot… and it becomes troubling for [me]. - 28 year-old, male medic

The resource strain on medics and clinics constantly hindered care in IDP areas. The interviewed medics spoke of the difficulties of under-resourced clinics, which struggled to deal with high patient volumes and diverse ailments, especially with mass casualties from conflict (n=10). They felt frustration, stress, and anxiety when they knew a patient’s diagnosis, but were powerless to treat the patient because of limited supplies.

The resource issues were further confounded by transportation difficulties between clinics and isolated villages (n = 3). Motor vehicles in mountainous jungle areas could not traverse dirt roads, trails frequently disappeared under dense overgrowth, and low areas were prone to flooding during rainy months. Due to these transportation barriers, medics struggled to deliver supplies or transport patients by foot, and patients were unable to solicit care in emergent situations. For example, a senior medic recounted guiding a trainee to perform a below-knee amputation for a landmine victim over the radio, because he was unable to cross a flooded river to do the operation himself.

While health organizations struggled to maintain the functioning of under-resourced and under-staffed clinics, their efforts were hindered by the frequent loss of staff, as several medics recounted (n=11). Medics related instances in which fellow medics left their jobs for other financial opportunities (returning to work on their farm, or setting up small businesses in their villages). In other cases, individuals left to become KNU soldiers or resettle to other countries as refugees.

### Personal/family-related challenges

I came to work here [as a medic] six years ago, but I [have seen] my family only two times. The first time was only [for] one hour, the second time, one day. - 25 year-old male medic

The majority of medics (n =24) seldom saw their families for extended periods of time, ranging from once every three months to over a decade. Although health organizations attempted to place medics in nearby or familiar areas, personnel constraints meant that the majority of medics had to work in villages far from their home communities, often requiring days or weeks to traverse by foot along un-maintained jungle trails. In some cases, medics’ families lived in refugee camps in Thailand or had resettled to other countries (n = 4). A minority of subjects communicated with relatives using mobile phones every few months.

The distance from home communities had disruptive effects on medics’ lives. Many felt unable to provide basic amenities or financial stability for their families (n = 8). Even though they were able to send money home to their families, some worried about their families’ security should they become disabled, die during service, or be captured by the Burmese military (n=3).

### Security issues, conflict, and human rights violations

Unanimously, security issues and threats of violence from the Burmese army were a major stressor for the medics. Inevitably, all medics found themselves working in unstable security situations at some point during their career (n = 30).

These continuing security threats limited medics’ ability to provide health services (n = 9). Community health organizations often deployed medics to villages in close proximity to active fighting. Several medics described fleeing villages under attack and having to abandon patients – a source of frustration and feelings of futility. One medic recounted when Burmese soldiers besieged their village and clinic with mortar fire for five days.

Medics and their clinics were vulnerable to attacks from military patrols, especially when transporting patients through jungle areas between villages (n =10). Medics typically traveled alone or in small groups of two or three medics without armed escorts. One medic reported being ambushed by a group of Burmese soldiers, forcing him to hide with the patient and return fire to defend himself. One army medic recounted a story of being shot at while crossing a river with supplies:

…When [the medics] crossed the river… they [stood] on the bamboo boat and they tried to cross the river… and when they crossed, the Burmese army shot at them –after that, [the medics] jumped down into the river and tried to swim across … - 21 year-old, female medic

The fear of being attacked while traveling was a significant source of anxiety; one medic claimed to search for escape routes in the forest when stationed at a village, even in times of relative stability.

Additionally, medics were often at risk of injury from antipersonnel landmines, with many reportedly triggering or nearly detonating landmines (n = 8). One subject reported an instance where a fellow medic was injured after stepping on a landmine, requiring him to carry the wounded medic back several miles to be treated:

On the road, one of my friends stepped on a landmine, and he [needed to] get amputated… We had to [stop] to control bleeding. That area is very close [to fighting between] the [Burmese] and KNU troops.

I tore my *longyi* [sarong-like cloth] in order to pack and compress the injury… I carried him on my back for three hours… but it was too much bleeding.

We arrived at the clinic, and gave a blood transfusion… two bags of blood. And we began an upper knee [amputation]. I was very worried and… so afraid at that time. He is my friend – I worried about… for their family, their home. - 30 year-old, male medic

Mobile clinics were also targeted by troops and were sometimes destroyed or burned when discovered, displacing both medics and their patients. One medic reported that his clinic had to be rebuilt numerous times after subsequent attacks. In addition, medics were often forced into hiding along with the displaced populations after attacks on villages (n = 4).

### Early life trauma

During interviews, medics were asked to recall any traumatic experiences prior to working as a medic. Several medics recounted being forcibly displaced from their village at an early age (n=10). One medic told the story of how her mother and sisters died while hiding in the jungle following displacement:

…When I was seven years old, [my family] had to [flee our village when it was under attack]. [My sisters and I] were only children… we could not run together with the adults, so my father had to carry us in his basket and we fled into the forest.

We could not live in a stable place, and sometime we [had no] food to eat… So we had to move around the jungle, always hiding. My mother, because she had to move like this, she [became sick] and no one checked… and there was no one to take care of her, so my mother died. Later on, my two little sisters also died. - 36 year-old, female medic

Accounts of displacement often involved violence against other individuals. A significant portion of medics had witnessed their relatives being beaten, tortured, or killed by Burmese soldiers (n = 7). One medic recounted how soldiers tortured and sexually assaulted his relatives in front of the rest of his family. Another medic recounted how his pregnant aunt was publicly physically assaulted. Another spoke of involuntarily re-experiencing an early traumatic experience in nightmares:

…When I was ten, the Burmese soldiers came and arrested my father and asked [if he owned a] gun. My father did not have a gun…[so the soldiers put] a knife to his back, to threaten [him]. Then they beat him.

It's [been] a long time… but, sometimes [I have] nightmares [of it]. … like somebody would come and stab [me] with a knife. I wake up, and I think ‘this is a dream’. I have heart palpitations, like I'm afraid, and… I am sweating when I wake up. - 25 year-old, male medic

Many medics drew upon their personal experiences of displacement as a source of empathy for their patients in IDP areas. These traumatic memories could be triggered when hearing patients’ similar experiences:

This kind of image, this kind of memory, it doesn’t haunt us – but we cannot ever forget. Usually we're trying to delete this kind of memory, but we still remember… We will remember sometimes when we see patients [with] gun injuries, we may remember these feelings. - 24 year-old, female medic

…When we go to [work] in the field, [we see] families and our people who escaped and are living, hiding… I think always – I used to hide like this. I used to move like that. - 23 year-old, female medic

The connection to patients that medics felt when serving war-affected villages was a crucial motivational factor that will be discussed below.

### Support and management of stressors

Medics managed their distress largely through a combination of 1) peer group interactions, 2) various forms of coping mechanisms, and 3) goal formation and motivation.

#### ***Peer-group interactions***

Many subjects felt that the relations developed within medic teams were essential for psychological wellbeing and social support, especially in isolated or unstable areas (n =11). Some medics characterized a strong mentor-apprentice relationship between experienced and novice medics (n =7). Since medics reported learning the bulk of their skills in field practice, a sense of peer camaraderie and dedicated mentorship was crucial for professional development (n=2).

However, some medics (n= 4) reported poor communication of work-related issues or emotional support amongst their team. The reluctance of medics to reveal their personal difficulties seemed to be multi-factorial, stemming from individual's discomfort with expressing their feelings as well as social norms that discouraged sharing emotions or admitting weakness. These medics expressed a perceived feeling of isolation, low confidence, and difficulty coping with work stressors.

### Forms of coping mechanism

In the interviews, medics were encouraged to give examples of strategies and activities used to cope with stressors. Forms of coping included confiding in friends and family, group activities such as sports or singing, and personal activities such as meditating, hunting, and swimming.

Religion, in the form of attending church and choir singing, was an important source of community support and social bonding. Though not all Karen medics were Christian, individuals working for the KNU (as medics or otherwise) were more likely to be Skaw Karen-speaking, Baptist Christians [54]. Some medics (n = 4) cited religious activities, such as praying, as a major source of stress relief and motivation.

A minority of medics admitted to using alcohol or narcotic substances to cope with stressors (n= 2). Abused drugs included benzodiazepines, chlorpheniramine, propanolol, and morphine – all medicines carried by medics for therapeutic purposes. Some respondents reported of medics who were unable to cope with their difficulties and committed suicide.

### Goals and motivations

The majority of medics (n=18) said that helping their communities by successfully treating patients was their primary motivation for starting or continuing their work. Two medics said that a powerful motivator was “feeling needed” by their leaders and village communities. Medics who were able to visit their families with relative frequency felt encouraged to continue work. Many medics shared career goals that they were progressing towards, including improving medical skills and attaining leadership roles in their organizations.

## Discussion

### Interpretation of quantitative and qualitative results

If we assume a conservative approach to threshold values, the observed survey means may indicate relatively low aggregate symptom scores of PTSD, anxiety and depression in this population. This is in contrast with the qualitative evidence of the burden of subjects' abundant life stressors, especially given that a third of interviewed medics had experienced displacement and all 30 medics had felt heavily threatened by security issues. There are several possible explanations for these results:

### Factors of resilience

It is possible that some medics exhibited a high degree of psychological resilience to help them cope despite abundant stressors, resulting in lower symptoms reporting. The respect granted to medics by their communities played an intrinsic role in promoting positive identity formation and developing self-efficacy over victimization. In contrast to the majority of forcibly displaced Karen individuals, the medics enjoyed significant empowerment to benefit their communities. When working in high-stress environments without strong family support, some medics’ teams provided essential social support through peer camaraderie. Finally, interacting with patients who underwent similar trauma may have allowed medics to better process their own experiences. This could have had a therapeutic or protective effect against developing symptoms of PTSD, anxiety, or depression.

### Discrepancy in demographics between quantitative and qualitative samples

There was concern during analysis that the less-experienced surveyed medic sample would display less severe manifestations of distress than the more experienced interviewed sample. Thirty-five percent of the quantitative sample consisted of medics in training, with less than a year of experience. The similarity in means between the sample population (n =74) and the experienced subset (>2 years of experience; n =42) may suggest that years of work experience may contribute less to psychological distress than other factors (such as early-life trauma), or that greater experience has a protective factor over repeated stressors. Further investigation must be done to substantiate these hypotheses.

### Study limitations

There are several limitations of this study worthy of elaboration:

### Potential inadequacy of symptomatic criteria of screening tools

Although the DSM IV criteria of depression and anxiety disorders (including PTSD) may be a helpful guide when screening individuals for mental health interventions, symptoms criteria of diagnostic frameworks must be approached cautiously. The GHQ-12 and PCL-C instruments used in this study may not have detected culturally specific signs and symptoms of mental illness in this setting (e.g. the physical manifestations of psychological distress presented in the *local idioms* section), resulting in lower mean survey scores despite true underlying psychiatric symptomatology.

Furthermore, despite the high literacy level of the study participants, the fact that surveys required written responses may have affected the accuracy of reporting. For the many subjects unfamiliar with mental health concepts, describing their own mental health symptoms in written format may have been more difficult than it would have been through conversation. Also, qualitative interviews were conducted following survey administration; interviews could have facilitated recall of symptoms in survey responses.

### Potential incongruence of PTSD framework

It is possible that the framework of PTSD may not be fully culturally appropriate for the Karen population, though the qualitative evidence strongly suggested the existence of PTSD symptoms for some individuals. Though it is recognized that PTSD can manifest from either a single-event or chronic trauma [18], many conflict-related PTSD accounts observe a subsequent return to a “normal” environment after the traumatic occurrence – such as a soldier returning to peaceful civilian life [3,15]. The majority of Karen medics in this study have only ever inhabited an environment of heightened exposure to distress and repeated traumatic events. Put simply, there may be nothing “post-” about these medics’ traumatic stress when they have only experienced conflict and instability as their baseline – though some individuals clearly manifested symptoms with on-going and repeated exposures.

### Lack of validation of quantitative tools in this population

Survey instruments were not validated in reference to a “gold standard” (such as clinical interviews) for this population. Furthermore, no estimates of PTSD or mental illness prevalence currently exist to help determine threshold values for this population. Although the PCL-C and GHQ-12 were both designed to be effective without adaptation to specific cultural settings, adapting and validating psychometric screening instruments (that explore culturally relevant manifestations of mental illness and use accurate idioms to describe symptoms) would improve effectiveness for local contexts.

### Non-random sampling of surveyed subjects

The quantitative results of this pilot study should be interpreted cautiously given the small, non-random study sample with restricted representation of the larger population of medics. Due to the inherent limitations in subject recruitment and sampling, the authors had very limited ability to control for dependent variables in the quantitative analysis.

These limitations likely also affected the sample’s representation of qualitative evidence. The medics attending trainings perhaps had stronger career motivations than those who did not attend. Given their presence at training courses, medics may have given responses that reflect their organizations’ goals and intentions over their personal views. Furthermore, interviewing and surveying medics far from their communities and work environments may have biased their responses via emotional distancing. For the reasons stated above, the results of this study may have limited generalizability to populations of health providers working in other regions of Myanmar, or medics in other conflict areas worldwide.

### Inability to correlate qualitative evidence of stressors with symptom scores

This study was not designed to distinguish the effects of different sources of mental distress as measured by the quantitative scales. For example, although one’s previous history of personal trauma can have significant impact on the development of symptoms [56], the effects of early life trauma could not be delineated from other factors contributing to PTSD, depression, and anxiety symptoms among the study subjects. Furthermore, the qualitative evidence of drug and alcohol abuse was elicited after the quantitative survey was designed, and thus this topic was not systematically explored.

### Cultural and linguistic barriers

Finally, the use of an English-Karen interpreter for most of the interviews may have misrepresented or biased subjects’ responses. Ideally, the investigator in future studies should communicate in local languages.

### Study strengths

This study of medics in Karen state, eastern Myanmar utilized both quantitative and qualitative methods to identify stressors and evaluate manifestations of mental distress amongst an understudied population of health providers in a low-resource conflict area. This is one of the few mental health studies conducted in eastern Myanmar and, to our knowledge, the first to focus exclusively on the psychological experiences and coping strategies of ethnic Karen community healthworkers. This study can be considered an initial platform from which to base further investigation of this population of medics, as well as a contribution to the wider literature of vicarious traumatization, burnout, and resilience amongst health providers and emergency responders in conflict and disaster settings worldwide.

## Implications

There are several implications for targeted mental health interventions and future research for this study population that can be applied generally to populations in conflict or post-conflict settings:

### Implications for the study population

The discrepancy between quantitative and qualitative findings suggests that the PCL-C and GHQ-12 scales should be culturally adapted and validated for the study population in future studies. However, the significant correlation between PCL-C scores and the four-point GHQ-12 scores suggests that using both instruments can corroborate screening to better identify individuals for targeted interventions.

Starting in 2008, annual mental health support trainings have been piloted for KDHW trauma management medics, and these trainings will be expanded to other medic teams in 2013. The goal of the workshops have been to train medics to address mental health concerns for their patients, but also to provide medics with techniques for their own and their peers’ mental wellbeing.

### General implications for healthworkers in low-resource conflict settings

The qualitative evidence corroborates previous literature that community, collaboration, and cooperation amongst coworkers protect against professional burnout and vicarious traumatization among care providers and emergency first-responders [10,57], and that clarity of team roles and the social support received by colleagues and superiors promote positive work engagement [58]. Without a supportive work environment, care providers can be at greater risk for secondary trauma, compassion fatigue and burnout [59], as indicated by the low confidence and coping ability amongst the medics with poor team support. This study promotes the idea that mental health trainings for care providers in emergency settings can aid in developing emotional self-care and peer support, in addition to teaching skills to manage patients’ psychological distress. In mental health training seminars, the PCL-C and GHQ-12 may be useful as training tools to assist medics in discussing depression, anxiety, and PTSD amongst both patients and peers.

Mental health professionals working with medics in low-resource conflict areas must recognize the importance of community factors while simultaneously supporting individual psychosocial needs. Emphasis should be placed on longitudinal team-building exercises, improving interpersonal communication, and developing coping strategies that are targeted and specific to individual, family, and community needs. Feelings of inadequate medical skills should be discussed and addressed during technical trainings. Unhealthy forms of coping, such as alcohol, medication, and narcotics abuse, should be identified and addressed in peer discussion groups.

Studies involving subject interviews can provide evidence of violations of medical neutrality committed by parties to a conflict. The targeting of medics, clinics, and their patients constitutes a violation of medical neutrality and IHL according to the Geneva Conventions of 1949 [1]. Medic trainings may present opportunities for local and international human rights organizations to document violations of IHL.

## Further research and conclusions

Future studies in this population should examine larger, randomized samples and develop culturally adapted and validated screening instruments for the Karen medics. This study of health providers can help to inform a general population study of anxiety, depression, and PTSD for those living in Karen IDP areas. Given the qualitative evidence of substance abuse, future surveys should also screen for drug and alcohol abuse and dependence. Finally, the role of cultural and religious factors should be explored in greater depth through future qualitative investigation.

The medics of Karen state, despite enormous challenges and stressors, have nonetheless exhibited marked resilience in the face of trauma. However, further support is needed to maintain the psychological wellbeing of these providers. If they continue to be the only source of health services for large populations that would not otherwise have care, increased attention to medics’ mental health is critical to prevent burnout and retain these essential personnel. As these medics form a crucial pillar of strength for the communities inside Karen state, their individual needs for physical and emotional support must not be abandoned.

## Abbreviations

BPHWT: Back pack health worker team; DSM: Diagnostic and statistical manual of mental health disorders; GHQ: General health questionnaire; ICRC: International committee of the red cross; IDP: Internally displaced persons; IHL: International humantarian law; KDHW: Karen department of health and welfare; KNU: Karen national union; PCL: Posttraumatic checklist; PTSD: Posttraumatic stress disorder; SD: Standard deviation; UC: University of California.

## Competing interests

The authors declare that they have no competing interests.

## Authors’ contributions

AGL conceived the study design, conducted the literature review, prepared the survey instruments, conducted data collection including interviews, managed survey data, performed data analysis and interpretation, drafted and revised the manuscript. LS advised the study design, helped coordinate field data collection, and provided critical revisions to the manuscript. EKSO contributed to the study conception and design, facilitated field data collection, and assisted with data interpretation. DPJ advised the literature review, study design, data analysis, and provided critical revisions to the manuscript. All authors read and approved the final manuscript.

## Supplementary Material

Additional file 1 Interview Guide. Click here for file
